# Supplementary material for: CYCLON and NPM1 Cooperate within an Oncogenic Network Predictive of R-CHOP Response in DLBCL
Source: Cancers (Basel). 2021 Nov 24;13(23):5900. doi: 10.3390/cancers13235900 (PMC8656558; doi:10.3390/cancers13235900)
Supplement: Supplementary file 1 [file cancers-13-05900-s001.zip › cancers-1322878-supplementary.pdf]

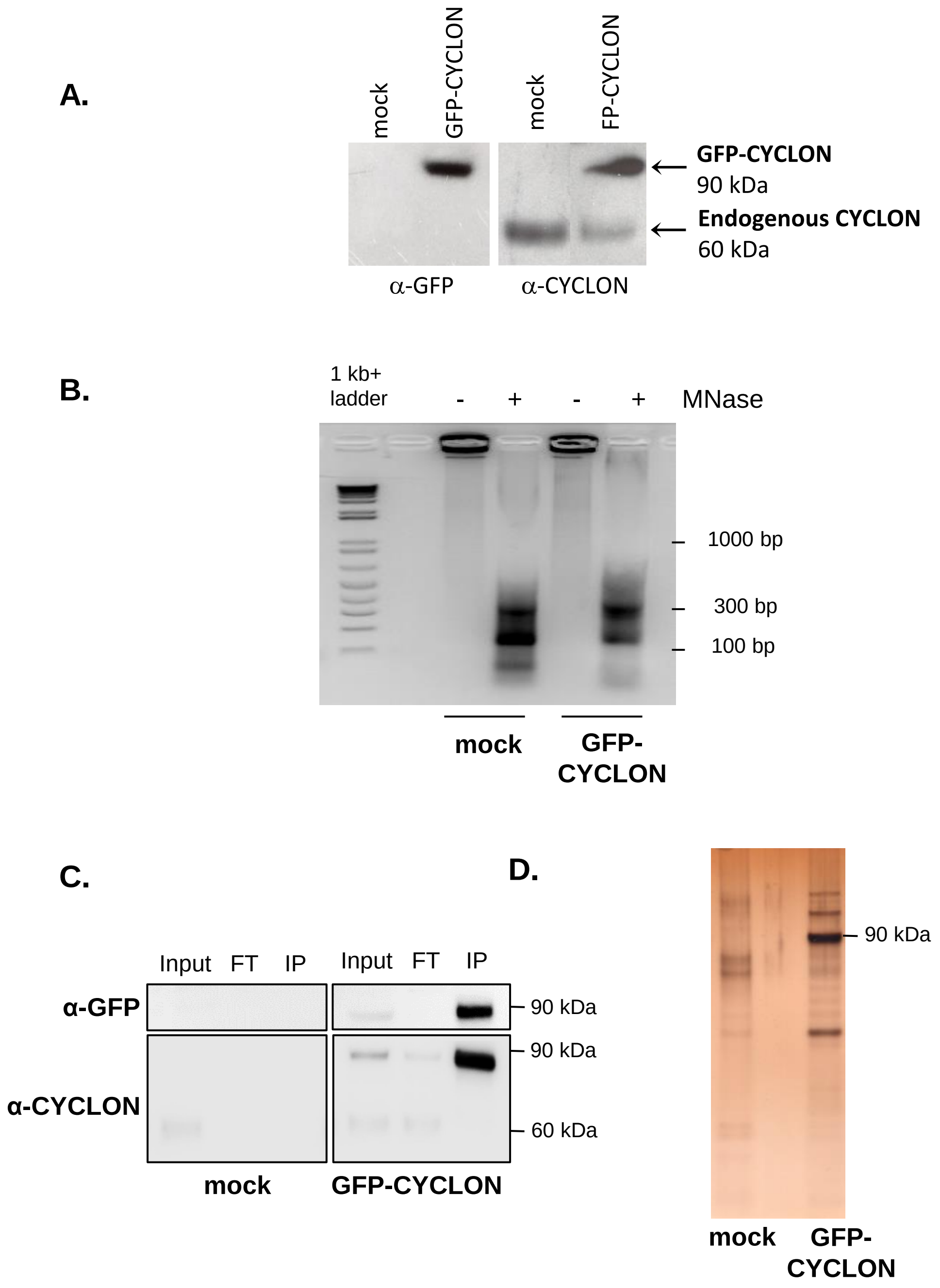

Figure S1: Experimental approach for identification of CYCLON protein-protein interaction network in B593 DLBCL cell line. A. Immunoblot analysis of B593 mock and GFP-CYCLON cells using anti-GFP and anti-CYCLON antibody. **B.** Agarose gel electrophoresis showing nucleic acids profiles before (-) and after (+) MNase digestion used to solubilize CYCLON-associated complexes. **C.** Western blot analysis of CYCLON and CYCLON-GFP in input, flow-through (FT, eq 1 volume) and GFP-Trap immunoprecipitated (IP, eq. 10 volumes) fractions as indicated. **D**. Representative silver staining of the GFP-Trap immunoprecipitated fraction in B593 mock and GFP-CYLON cell lines.

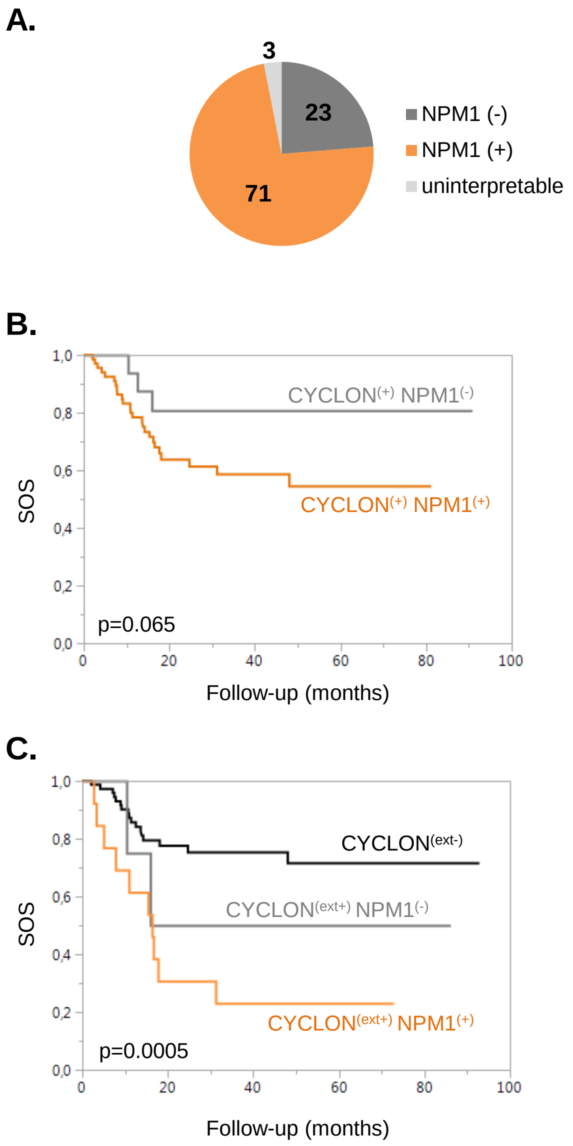

Figure S2: NPM1 IHC evaluation in DLBCL reveals an association of CYCLON/NPM1 double positivity with prognosis. A. Pie-chart representation of NPM1 positivity determined by IHC staining in a DLBCL cohort (n=97). **B**. Kaplan-Meier analysis of SOS associated with CYCLON single expressor (CYCLON$^{(+)}$ NPM1$^{(-)}$) or CYCLON/NPM1 double expressors (CYCLON$^{(+)}$ NPM1$^{(+)}$). **C.** Kaplan-Meier analysis of SOS associated with CYCLON non extra-nucleolar (CYCLON$^{(ext-)}$), CYCLON extra-nucleolar pattern/NPM1 negative (CYCLON$^{(ext+)}$ NPM1$^{(-)}$) or CYCLON extra-nucleolar pattern/NPM1 double expressors (CYCLON$^{(ext+)}$ NPM1$^{(+)}$). p values are derived from a log rank test.

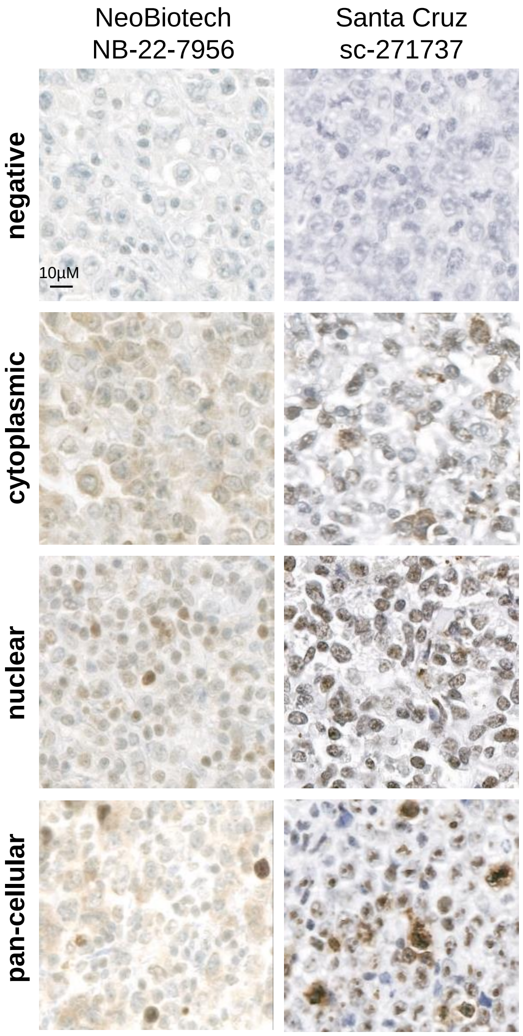

Figure S3. Validation of NPM1 staining patterns using an alternative antibody. IHC analysis performed on 26 DLBCL cases using 2 distinct mouse monoclonal antibodies (NeoBiotech clone 7H10B9 #NB-22-7956 and Santa Cruz #sc-271737) gave similar results, confirming the validity of NPM1 alternative localizations in DLBCL. Four representative cases are presented.

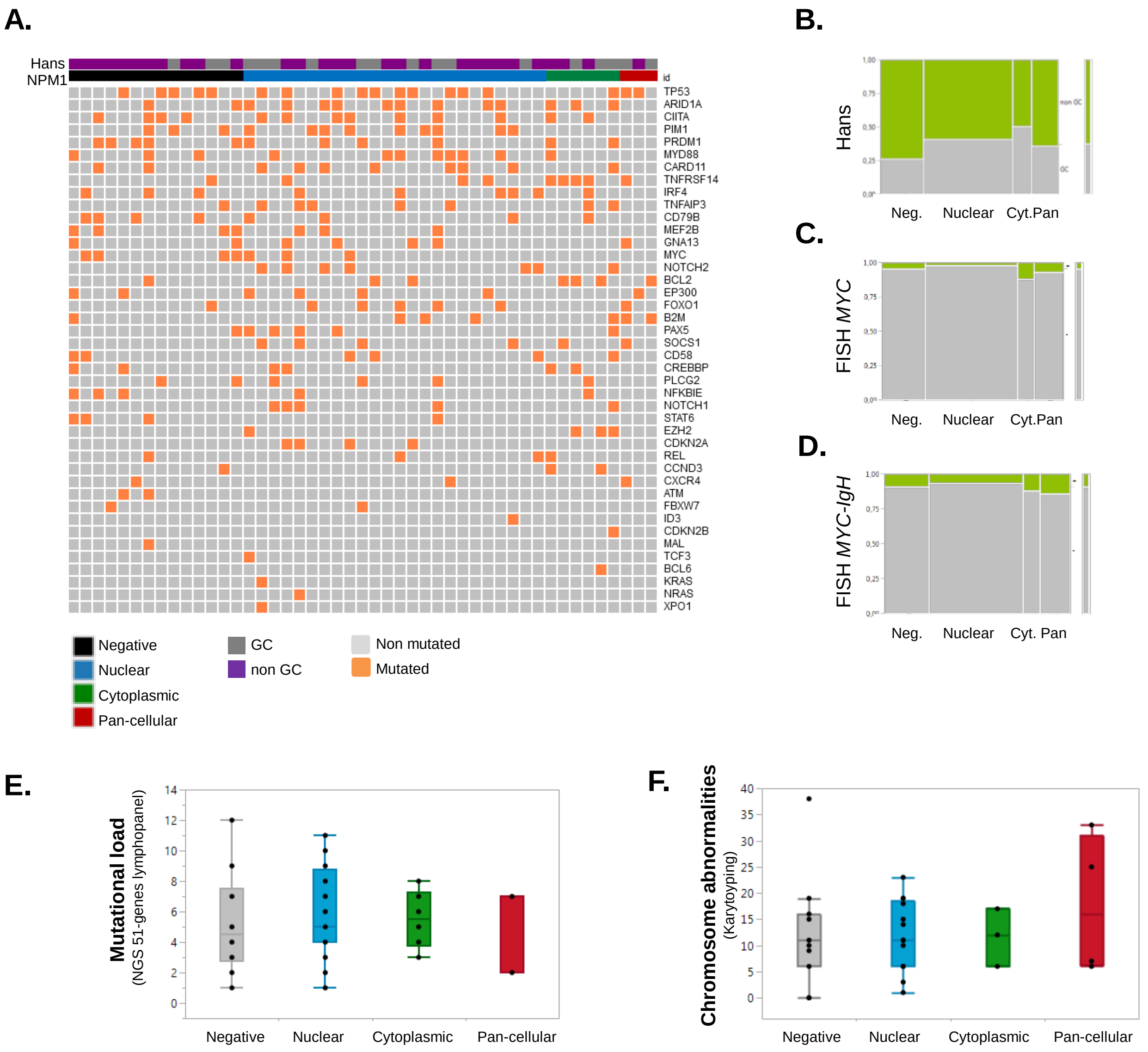

Figure S4. Investigation of genetic abnormalities according to NPM1 staining patterns. A. Heatmap representation of mutational profiles derived from NGS targeted sequencing (n=48) including 51 genes sorted by mutation frequency as indicated. **B-D.** Mosaic plots between NPM1 staining pattern and Hans non-GC/GC classification (B, n=97), *MYC* rearrangement (C, n=87), *MYC-IgH* rearrangement (D, n=87). **E-F.** Boxplot representation of mutational load (E, n=47) and number of chromosome abnomalities (F, n=38) for each NPM1 staining patterns.

A.

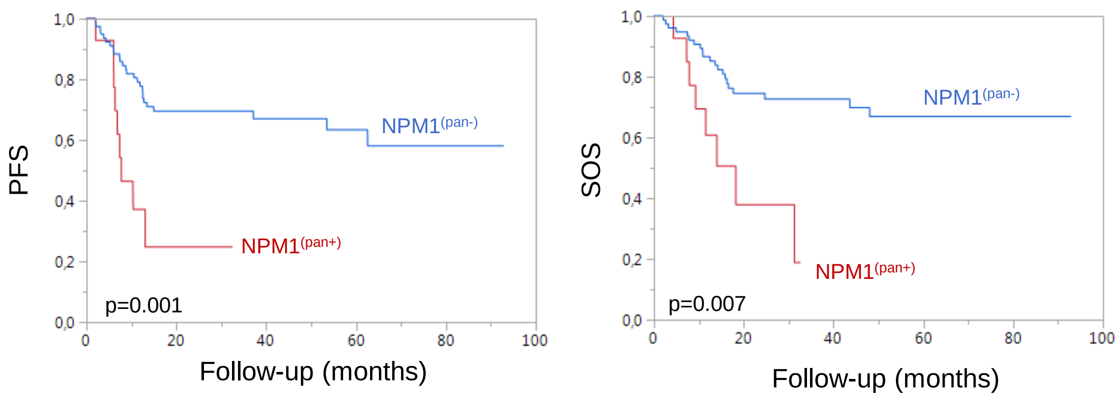

B.

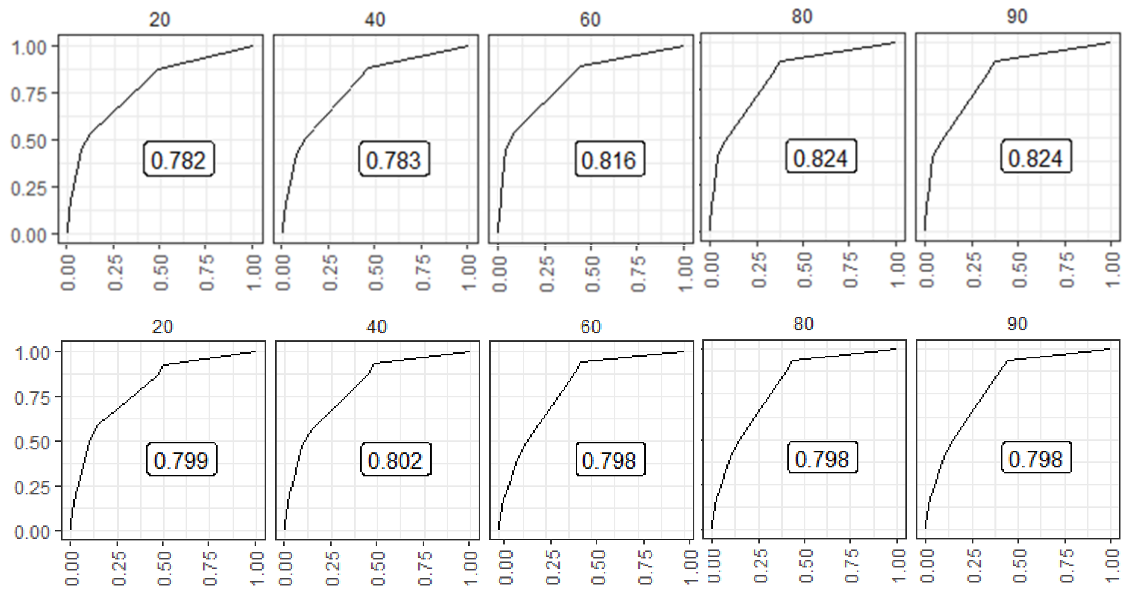

Figure S5: NPM1 pan-cellular staining (NPM1$^{(pan+)}$) defines a very poor prognosis group among DLBCL patients. A. Kaplan-Meier analyses of PFS (left) and SOS (right) associated with pan-cellular NPM1 staining (NPM1$^{(pan+)}$) and non pan-cellular NPM1 staining (NPM1$^{(pan-)}$). **B.** Time-dependent receiver operating characteristic (ROC) curves evaluating prediction accuracy of multivariate bootstrap Cox regression analysis of NPM1, CYCLON and R-IPI (Table 2) over time (20, 40, 60, 80 and 90 months as indicated). x axis: false positive rate, y axis: true positive rate. Box: area under the curve (AUC) for multivariate PFS (upper panel) and OS (lower panel) models.

Original_western_blot

Réf: BioRad
161-0374

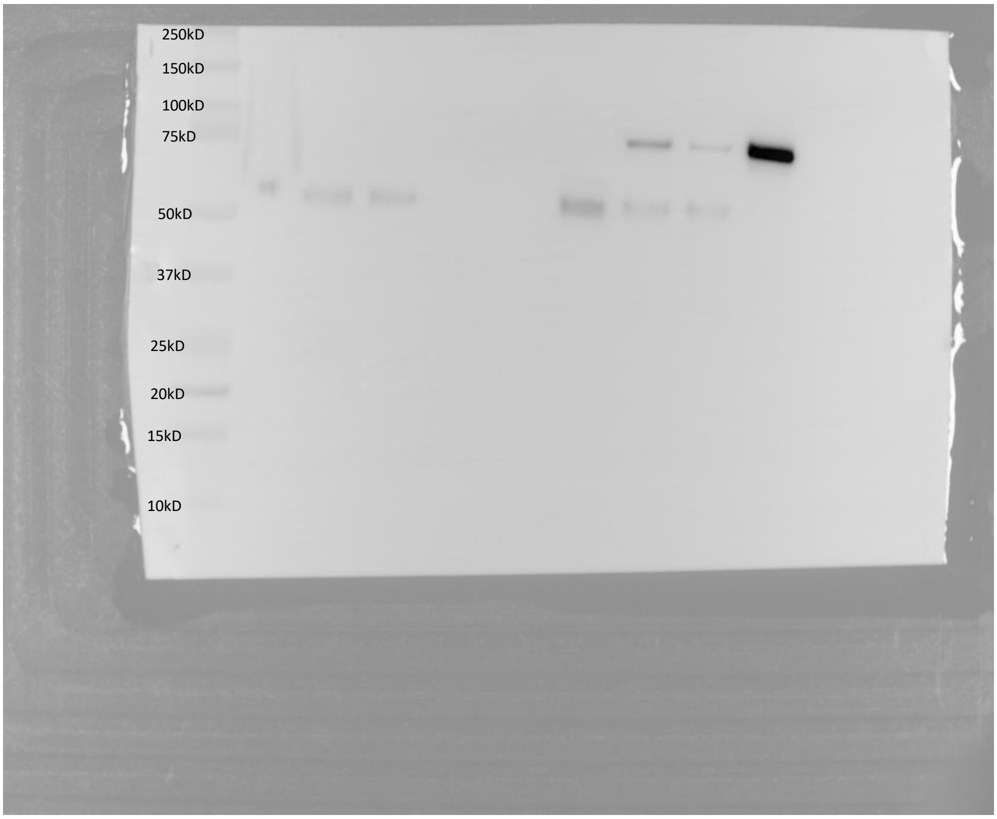

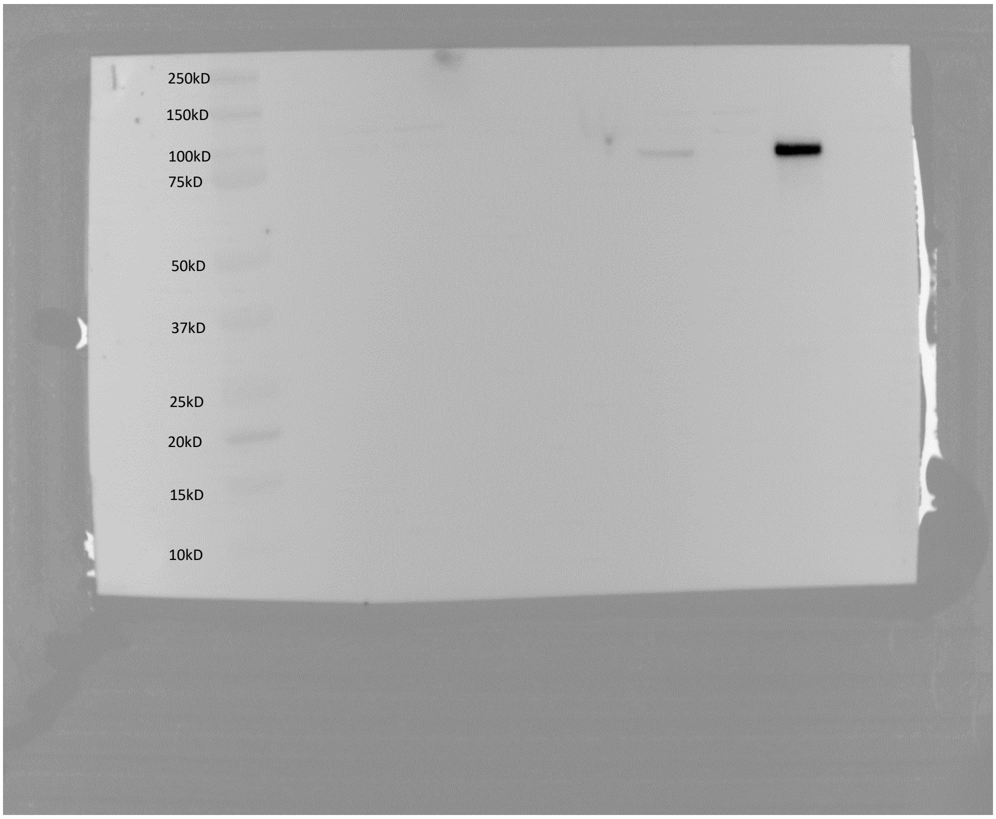
250kD
150kD
100kD
75kD
50kD
37kD
25kD
20kD
15kD
10kD

Table S1 : CYCLON interactome in B593 cells

This table lists proteins found significantly more abundant (log_2(Fold Change) ≥ 1.5 ; p-value ≤ 0.05) in eluates from GFP-Cyclon compared to control immunoprecipitations.

accession	protein name	gene name	Mol. Weight	Identified peptides	coverage (%)	log_2(Fold Change CYCLON/CTL)	p-value	abundance CTL1	abundance CTL2	abundance CTL3	abundance Cyclon1	abundance Cyclon2	abundance Cyclon3
**CCD86_HUMAN**	**Coiled-coil domain-containing protein 86**	**CCDC86**	**40236**	**21**	**50.28**	**11.57**	**2.61E-08**	**14.84335724**	**14.85226645**	**14.71945751**	**26.82049972**	**27.02233966**	**25.27892001**
B1B1J6_HUMAN	Chemokine-like protein TAFA-5 (Fragment)	TAFA5	19689	1	4.97	7.15	4.20E-04	14.84335724	14.85226645	14.71945751	24.6809757	22.87983925	18.29600344
DHX9_HUMAN	ATP-dependent RNA helicase A	DHX9	140958	32	24.65	6.52	1.12E-04	19.05211055	19.31978048	20.0429251	28.53570988	24.97127383	24.47450116
B3KTW4_HUMAN	cDNA FLJ38868 fis, clone MESAN2013211, highly similar to Protein FAM98A	FAM98A	37260	1	2.25	5.58	1.14E-03	14.84335724	14.85226645	14.71945751	22.71550264	21.24375906	17.20094317
Q59EC0_HUMAN	Adenosine deaminase, RNA-specific isoform ADAR-a variant (Fragment)	DSRAD	137833	21	17.68	5.51	9.47E-04	19.23409719	15.33922293	18.67450145	25.20755392	22.27234265	22.29081667
ALBU_HUMAN	Serum albumin	**ALB***	69367	18	25.62	5.40	1.19E-03	21.8966324	21.40461257	23.72059441	29.87569185	28.29568509	25.04383951
U2AFM_HUMAN	U2 small nuclear ribonucleoprotein auxiliary factor 35 kDa subunit-related protein 2	ZRSR2	58045	1	1.66	4.98	1.45E-04	14.84335724	14.85226645	14.71945751	20.91474523	20.26520537	18.17025965
RT22_HUMAN	28S ribosomal protein S22, mitochondrial	MRPS22	41280	4	9.17	4.92	2.81E-04	15.0717662	15.4782971	14.53293325	18.30176323	21.55375815	19.9767098
ROA0_HUMAN	Heterogeneous nuclear ribonucleoprotein A0	HNRNPA0	30841	1	5.57	4.56	9.44E-05	14.84335724	14.85226645	14.71945751	19.05011822	18.66801153	20.37442415
5NT1B_HUMAN	Cytosolic 5'-nucleotidase 1B	NT5C1B	68804	1	1.31	4.49	3.49E-02	14.84335724	14.85226645	14.71945751	22.82586426	21.06170345	14.00296406
TRIM5_HUMAN	Tripartite motif-containing protein 5	TRIM5	56338	1	1.42	4.11	1.13E-03	16.0500409	17.5703621	16.14148191	21.5901322	21.53946827	18.95034607
ILF3_HUMAN	Interleukin enhancer-binding factor 3	ILF3	95338	8	8.28	3.62	5.89E-03	18.30047604	18.05094648	17.15730549	23.83979558	19.78927529	20.73779232
PTCD3_HUMAN	Pentatricopeptide repeat domain-containing protein 3, mitochondrial	PTCD3	78550	2	2.61	3.49	2.94E-03	14.84335724	14.85226645	14.71945751	16.40070981	19.55047175	18.9320219
RALY_HUMAN	RNA-binding protein Raly	RALY	32463	2	5.56	3.27	1.84E-03	16.86690815	14.96208474	17.10159095	19.72018243	19.42733062	19.60235327
NCOA5_HUMAN	Nuclear receptor coactivator 5	NCOA5	65536	4	7.43	3.25	1.25E-02	18.00318141	17.58127188	14.70730157	21.67939816	19.34718217	19.00977799
SSBP_HUMAN	Single-stranded DNA-binding protein, mitochondrial	SSBP1	17260	3	21.62	2.95	3.54E-03	16.94688099	18.60620875	16.77132937	20.97500645	20.43971944	19.76546454
RL11_HUMAN	60S ribosomal protein L11	RPL11	20252	3	16.29	2.78	4.29E-03	18.32091105	17.12476226	18.79563625	21.59057075	20.51693802	20.47023662
HNRH2_HUMAN	Heterogeneous nuclear ribonucleoprotein H2	HNRNPH2	49264	5	14.25	2.73	3.06E-02	20.44923474	16.94117843	20.91502763	21.94169259	23.21112773	21.35494349
CSK21_HUMAN	Casein kinase II subunit alpha	CSNK2A1	45144	1	1.79	2.73	7.12E-03	13.84558736	14.21696396	13.34713151	17.96307582	15.9428699	15.68340796
CHAP1_HUMAN	Chromosome alignment-maintaining phosphoprotein 1	CHAMP1	89099	4	5.67	2.49	4.20E-02	17.89212686	17.36651422	16.99545351	22.51099737	18.70766506	18.4938012
NPM_HUMAN	Nucleophosmin	NPM1	32575	11	40.82	2.41	4.05E-02	22.97270302	19.58807703	22.55617841	25.38420401	23.30362209	23.65071285
YLPM1_HUMAN	YLP motif-containing protein 1	YLPM1	219985	3	2.41	2.41	1.87E-02	16.99543643	16.93687184	16.92424308	20.74003865	17.70314188	19.63437325
HNRPC_HUMAN	Heterogeneous nuclear ribonucleoproteins C1/C2	HNRNPC	33670	9	27.12	2.37	8.08E-03	21.8359899	20.93343331	22.33927475	23.93934899	23.65740443	24.63047657
HNRH3_HUMAN	Heterogeneous nuclear ribonucleoprotein H3	HNRNPH3	36926	6	19.08	2.34	4.92E-02	19.36035324	16.68450736	20.58117744	20.57301908	20.69349127	22.3880408
RM38_HUMAN	39S ribosomal protein L38, mitochondrial	MRPL38	44597	1	2.63	2.24	1.75E-02	14.84335724	14.85226645	14.71945751	15.85904084	18.30247299	16.96138363
RM50_HUMAN	39S ribosomal protein L50, mitochondrial	MRPL50	18325	2	15.19	2.18	1.57E-02	15.8967047	16.32832488	15.33480242	17.41321501	19.09088203	17.59929303
KHDR1_HUMAN	KH domain-containing, RNA-binding, signal transduction-associated protein 1	KHDRBS1	48227	2	5.42	2.13	1.81E-02	18.5462055	16.55495408	16.96493023	19.72022887	19.41293331	19.33193009
RT27_HUMAN	28S ribosomal protein S27, mitochondrial	MRPS27	47611	2	4.83	2.09	3.01E-02	18.24532131	16.15215068	17.77884251	18.54859064	20.31313386	19.59284667
NH2L1_HUMAN	NHP2-like protein 1	SNU13	14174	3	25	2.04	3.25E-02	17.55394951	16.21566771	17.42564215	20.04557047	17.78512403	19.49181917
RS14_HUMAN	40S ribosomal protein S14	RPS14	16273	2	15.89	1.97	4.30E-02	16.48732461	17.70212005	15.86502747	17.72218187	19.97174659	18.26068276
MATR3_HUMAN	Matrin-3	MATR3	94623	6	6.38	1.95	1.88E-02	19.95500585	18.61726312	19.27685771	21.25401284	21.45519384	20.97852671
HNRPU_HUMAN	Heterogeneous nuclear ribonucleoprotein U	HNRNPU	90584	4	5.82	1.84	2.13E-02	16.66066174	15.86923338	16.74380347	18.48122202	17.96977138	18.3454814
ARHGH_HUMAN	Rho guanine nucleotide exchange factor 17	ARHGEF17	221673	1	0.39	1.79	4.14E-02	18.69410592	18.81672094	17.76979465	20.82974159	20.69417241	19.11284309
IF4A3_HUMAN	Eukaryotic initiation factor 4A-III	EIF4A3	46871	4	8.52	1.73	3.70E-02	17.01552631	17.9476555	18.14697472	18.99669479	19.15455293	20.14176801

Legend :

imputed values for values totally missing in the condition

imputed value for partially missing value in the condition

*ALB was excluded from the final list for graphical representation and ontology analysis as a very likely contaminant protein
